# Supplementary material for: Small Molecules Attenuate the Interplay between Conformational Fluctuations, Early Oligomerization and Amyloidosis of Alpha Synuclein
Source: Sci Rep. 2018 Apr 3;8:5481. doi: 10.1038/s41598-018-23718-3 (PMC5882917; doi:10.1038/s41598-018-23718-3)
Supplement: Supplementary file 1 — Supporting Information [file 41598_2018_23718_MOESM1_ESM.pdf]

SUPPLEMENTAL INFORMATION

Small Molecules Attenuate the Interplay between Conformational Fluctuations, Early Oligomerization and Amyloidosis of Alpha Synuclein

Sumanta Ghosh[!], Amrita Kundu[!], Krishnananda Chattopadhyay[*]

[!]Equal contribution

Protein Folding and Dynamics Laboratory, Structural Biology and Bioinformatics Division, CSIR-Indian Institute of Chemical Biology

Supporting Figure:

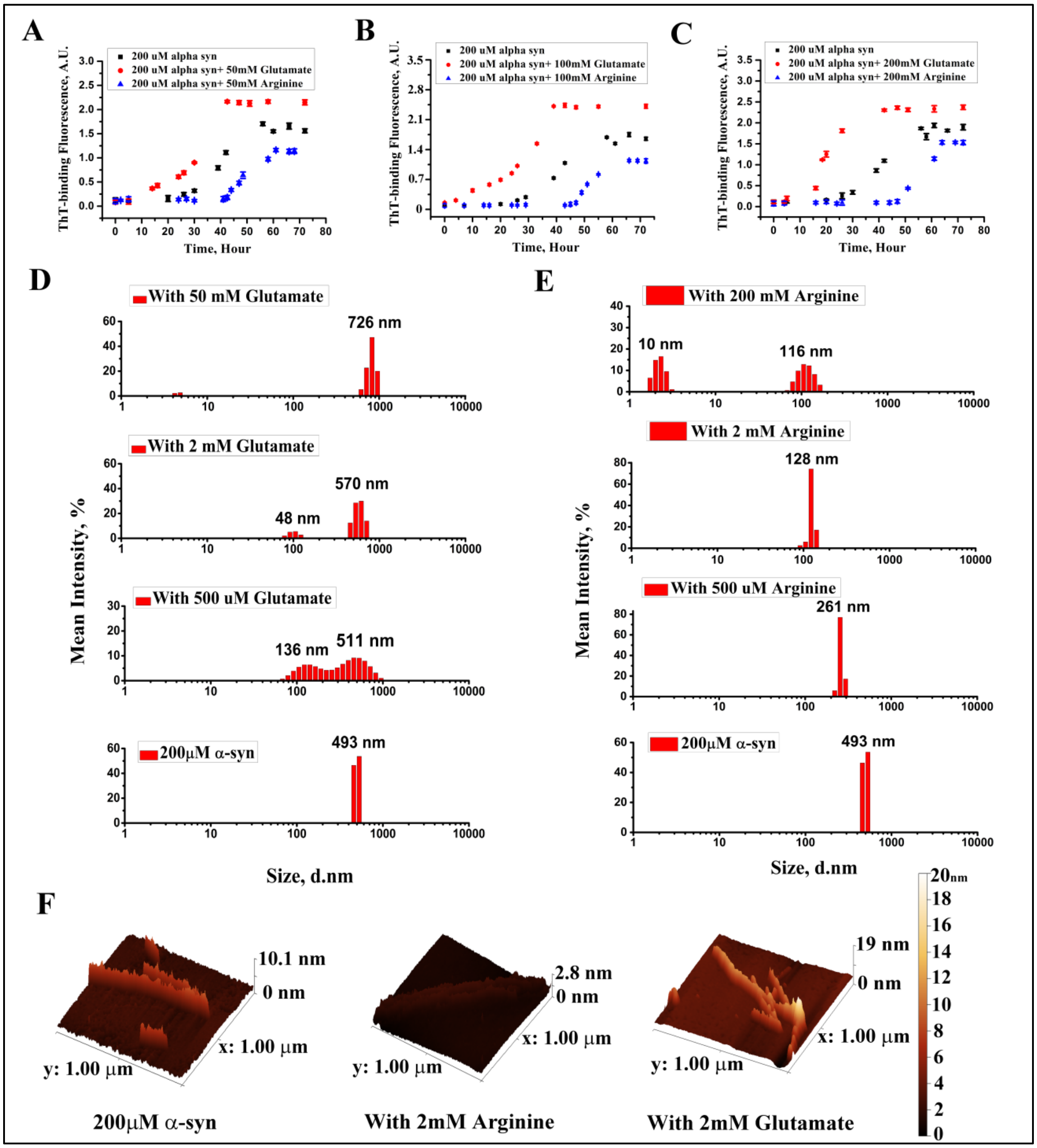

Figure S1: [A] Non-normalised ThT-binding fluorescence with 50mM, [B] 100mM and [C] 200mM arginine(blue) and glutamate(red) concentrations. ThT-binding fluorescence of wild type α-syn had been shown as a control (black) set of data in these figures; Data shown are mean±standard error. Error bars of ThT fluorescence experiments are calculated after repeating the experiments independently for eight times. [D] average hydrodynamic diameter (from DLS) with increasing concentrations of glutamate(red)and [E]arginine(black) from 50µM to 200 mM, [F] AFM images of α-syn fibrils at the saturated phase in the absence or presence of co-solvents. Colors represented the heights corresponding to the scale shown in the right.

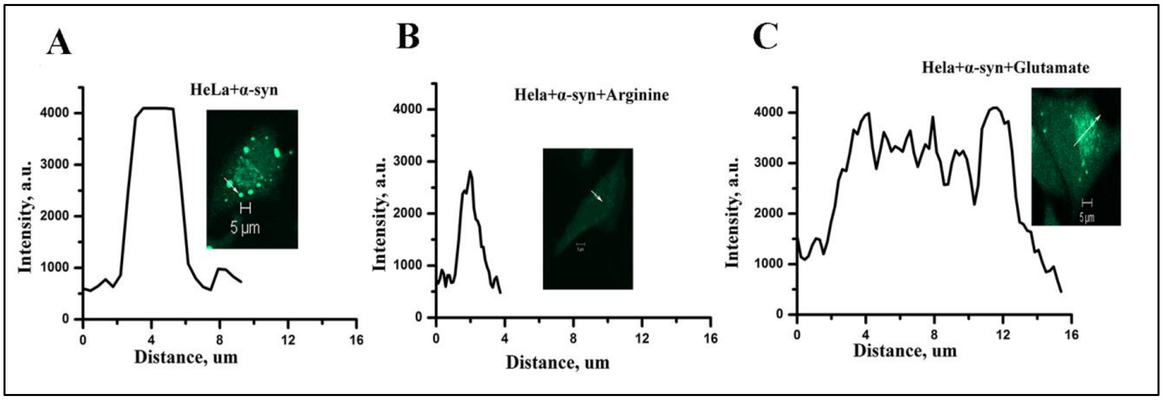

Figure S2: HeLa cells containing TC-AS aggregates [A] in the absence of any co-solvent, [B] in the presence of 500 μM arginine and [C] in the presence of 500μM glutamate. The intensity values calculated along the white arrows were plotted with distance. These analyses were done for 10 individual cells for each condition and one of them is shown above as a representative. Scale bars, 5μm.

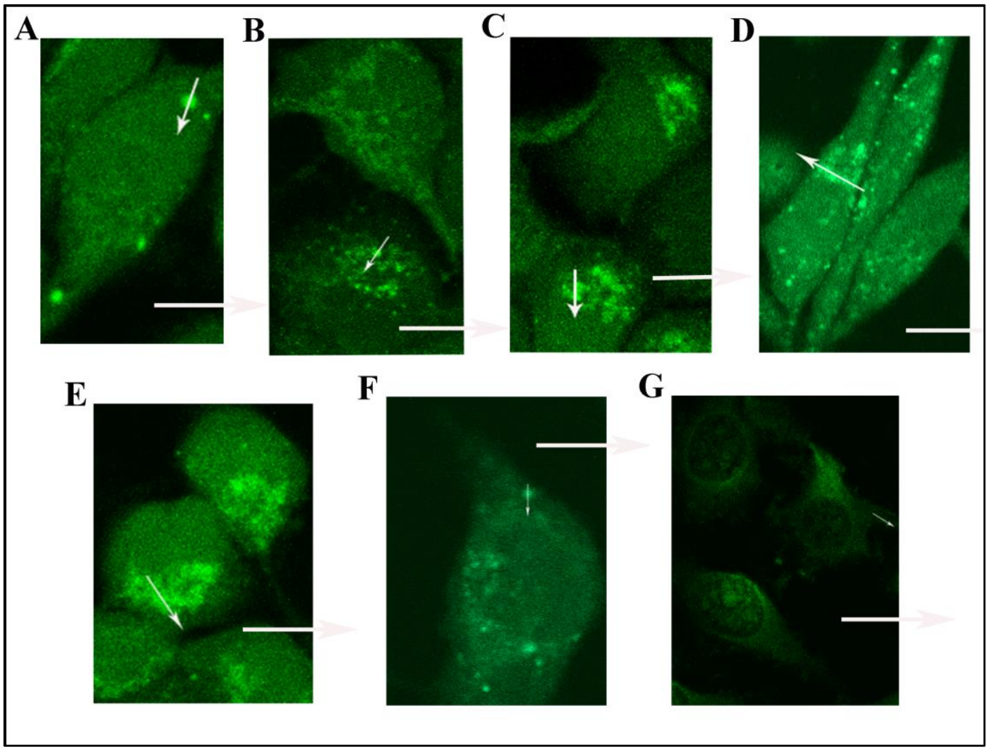

Figure S3: SH-SY5Y cells containing TC-AS in the [A] absence and presence of [B] Aspartate, [C] NaCl, [D] Glutamate, [E] Lysine, [F] Arginine and [G] Glutamate at a concentration of 500μM. The images were taken after 48 hours of transfection and with a 20 hours treatment with different small molecules. Scale bar 10μm.

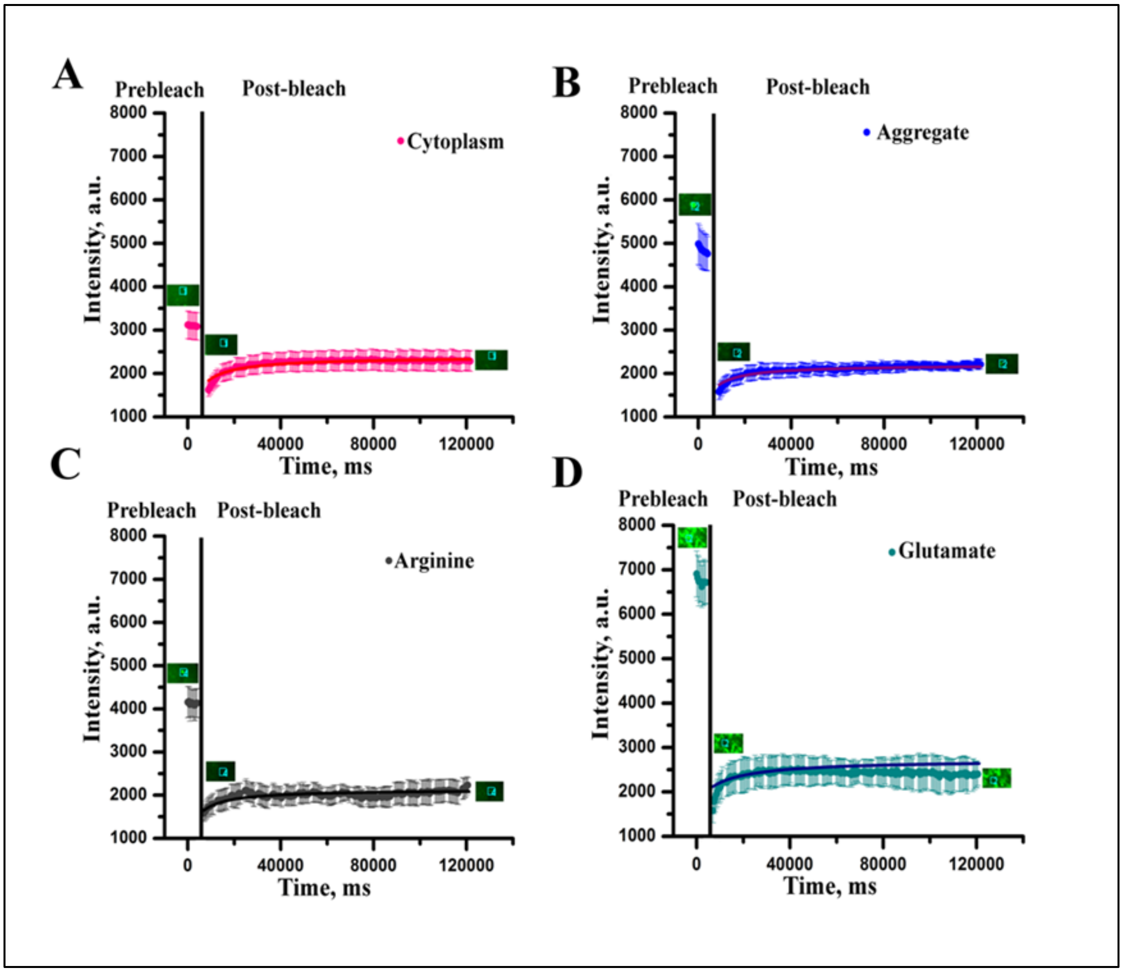

Figure S4: FRAP of TC-AS aggregates inside SH-SY5Y cells. Figure [A] and [B] represents non-aggregated and aggregated regions respectively; [C] and [D] represent FRAP of arginine and glutamate treated cell respectively. Normalized fluorescence intensities with corresponding ROIs are plotted with fits to a single exponential model. The insets show the images of the ROIs before photo-bleaching (0s), immediately after photo-bleaching (10s) and 120 seconds after photo-bleaching. Data shown are mean±standard error calculated after repeating the same experiments independently for three times.

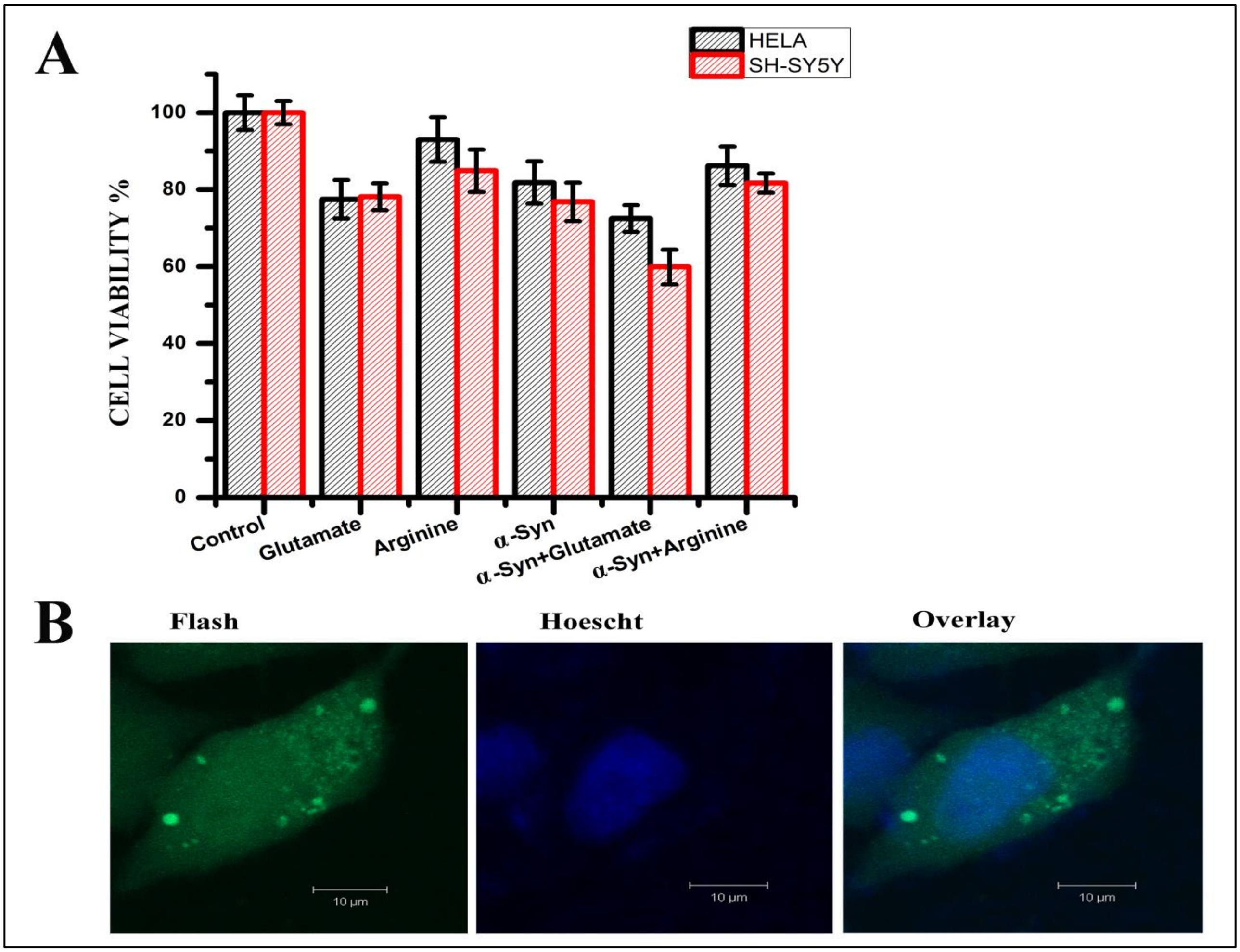

Figure S5: [A] MTT assay for HeLa and SH-SY5Y cells in differently treated conditions. Bars indicate average values of three independent experiments. Data shown are mean±standard error.[B] Nuclear staining with Hoechst shows SH-SY5Y cells in good health with an intact nucleus inside. The analysis performed on n=200 TC-AS overexpressing cells, one of them is shown above as representative. Scale bars, 10μm.

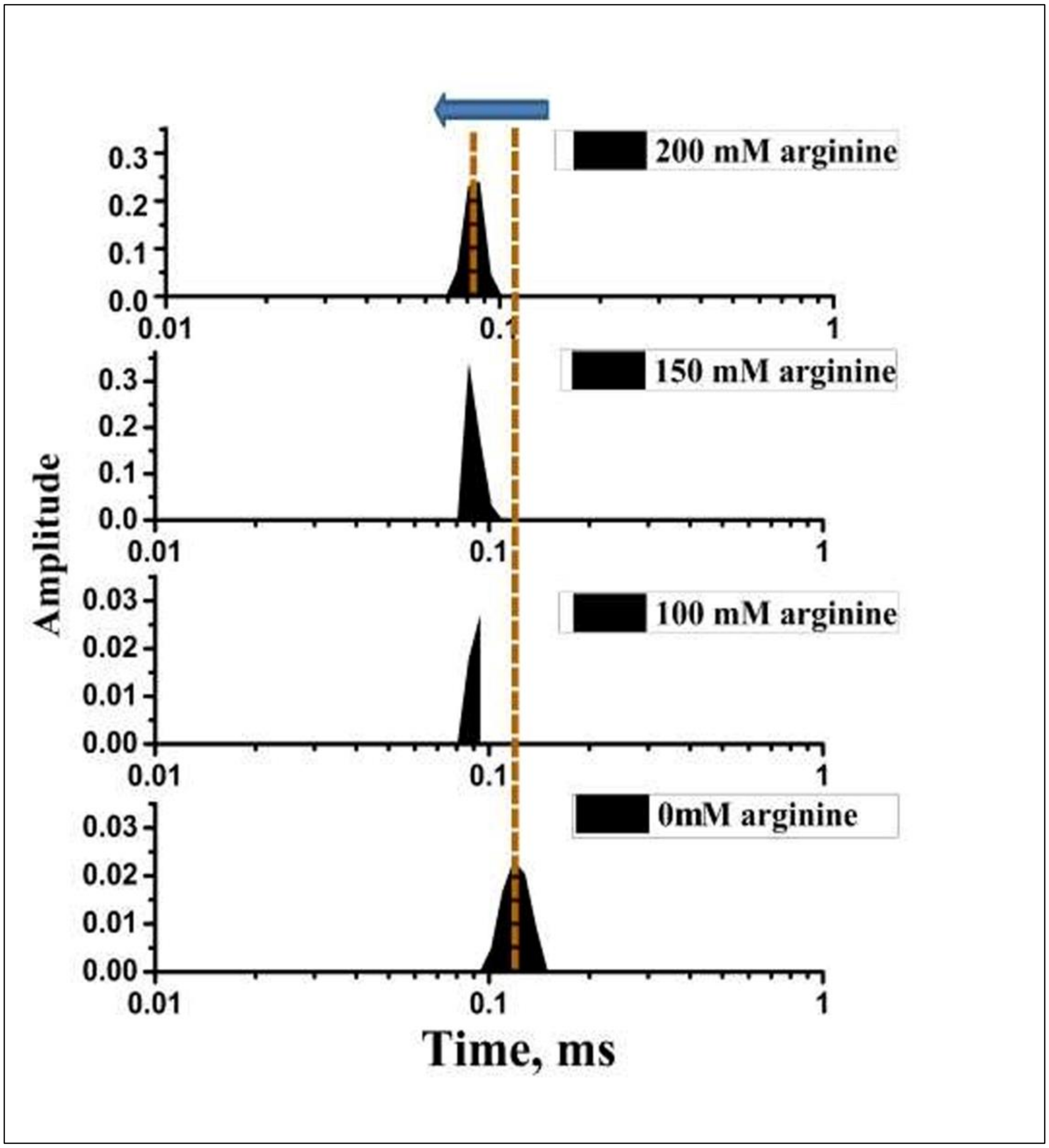

Figure S6: MEM FCS distributions in presence of different concentrations of arginine.

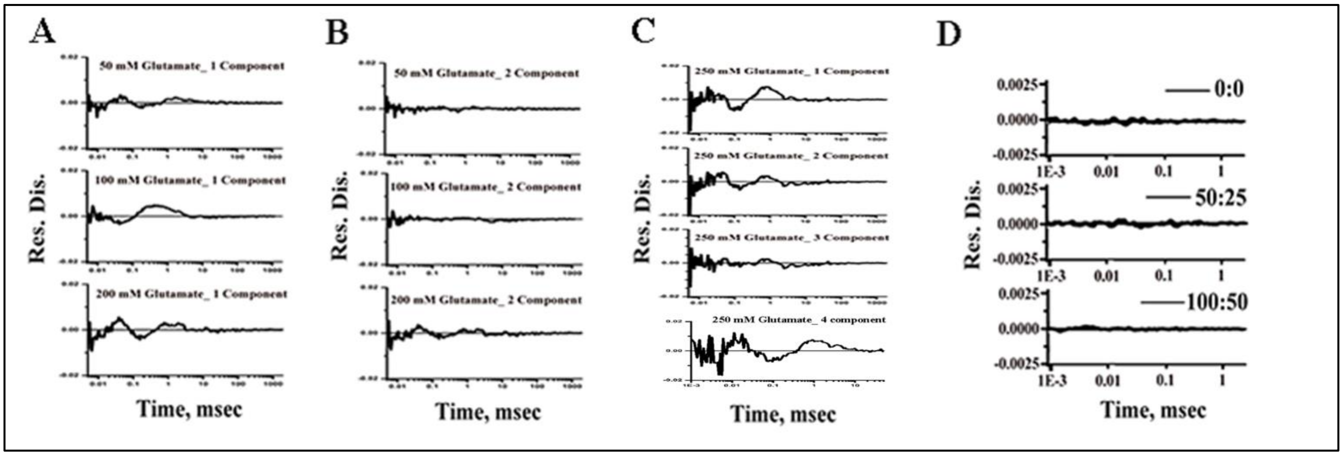

Figure S7: The residual distributions obtained from the FCS experiments in the presence of 50mM, 100mM, 200mM and 250mM glutamate. The number of components and glutamate concentrations are mentioned in each figure. The goodness of the fits is judged by the randomness of the residual distributions. As shown in figure [A], the use of one component model could not provide any reasonable fit in the presence of glutamate of any concentration (50mM, 100mM and 200mM). The use of a model containing two diffusing components fit the data well (figure [B]). As shown in figure [C], the addition of 250mM glutamate results in deviation from the two component model and at least a third component was necessary to obtain a reasonable fit. [D] Residual distributions of the two component diffusion analyses in the presence of different concentrations of arginine and glutamate keeping the ratio constant at 2:1. Error bars were calculated after five times of independent repetition of the experiments.

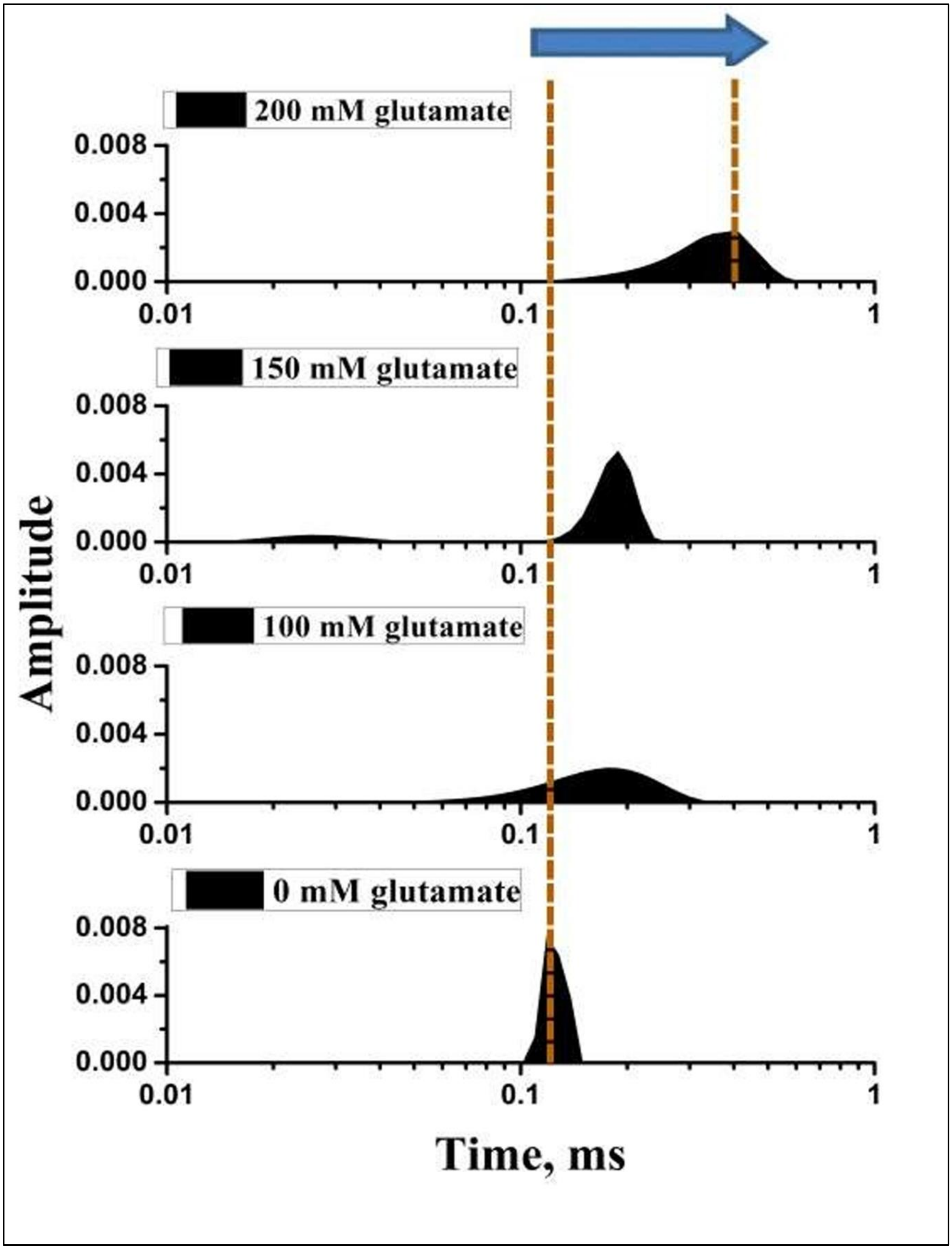

Figure S8: MEM FCS distributions in presence of different concentrations of glutamate.

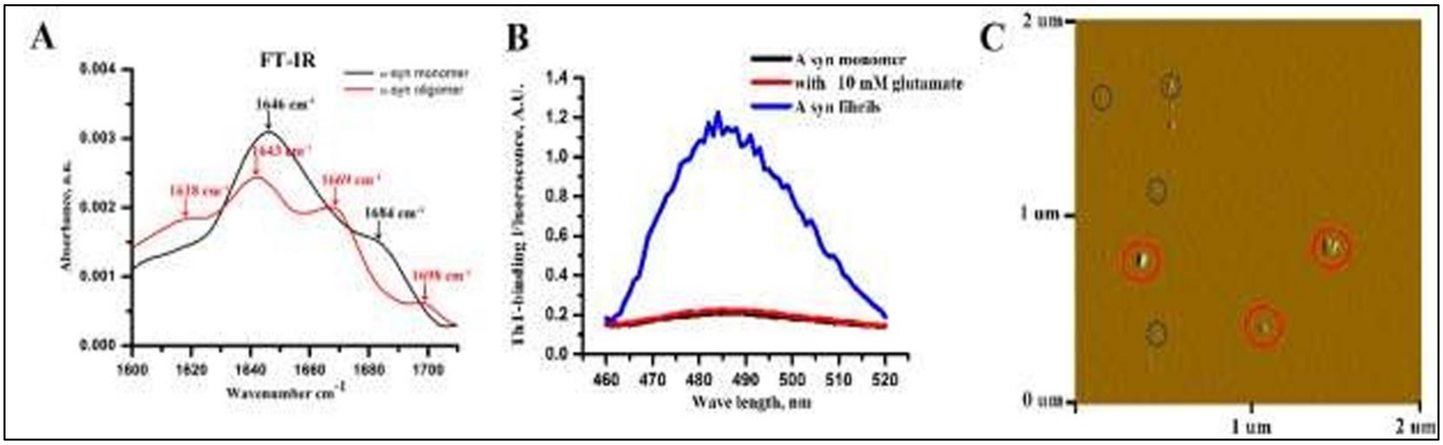

Figure S9: [A] FT-IR spectra of α-syn monomer (black) and oligomer (red, in the presence of 10mM glutamate). The amide I region of α-syn monomer showed two distinct peaks at 1646 and 1684 cm^{-1}. The first one represented random coil whereas the second peak was for β-turn. In the oligomer, the prominent appearance of 1698 cm^{-1} (for anti-paraller β-sheet) and 1618 cm^{-1} (for aggregated β-sheet) represented the starting of formation of oligomers and small aggregates. The other two peaks (1643 and 1669 cm^{-1}) were representative of random coil and β-turns respectively. [B] ThT fluorescence data of α-syn monomer(black), with 10 mM glutamate after 2 hours of incubation (red) and fibrils (blue). Presence of oligomers with 10 mM glutamate does not show significant ThT binding. [C] AFM image of α-syn with 10 mM glutamate after 3 hours of incubation. Red circle indicates larger oligomers (size 60-85 nm) and blue circle indicates small oligomers (size 20-35 nm).

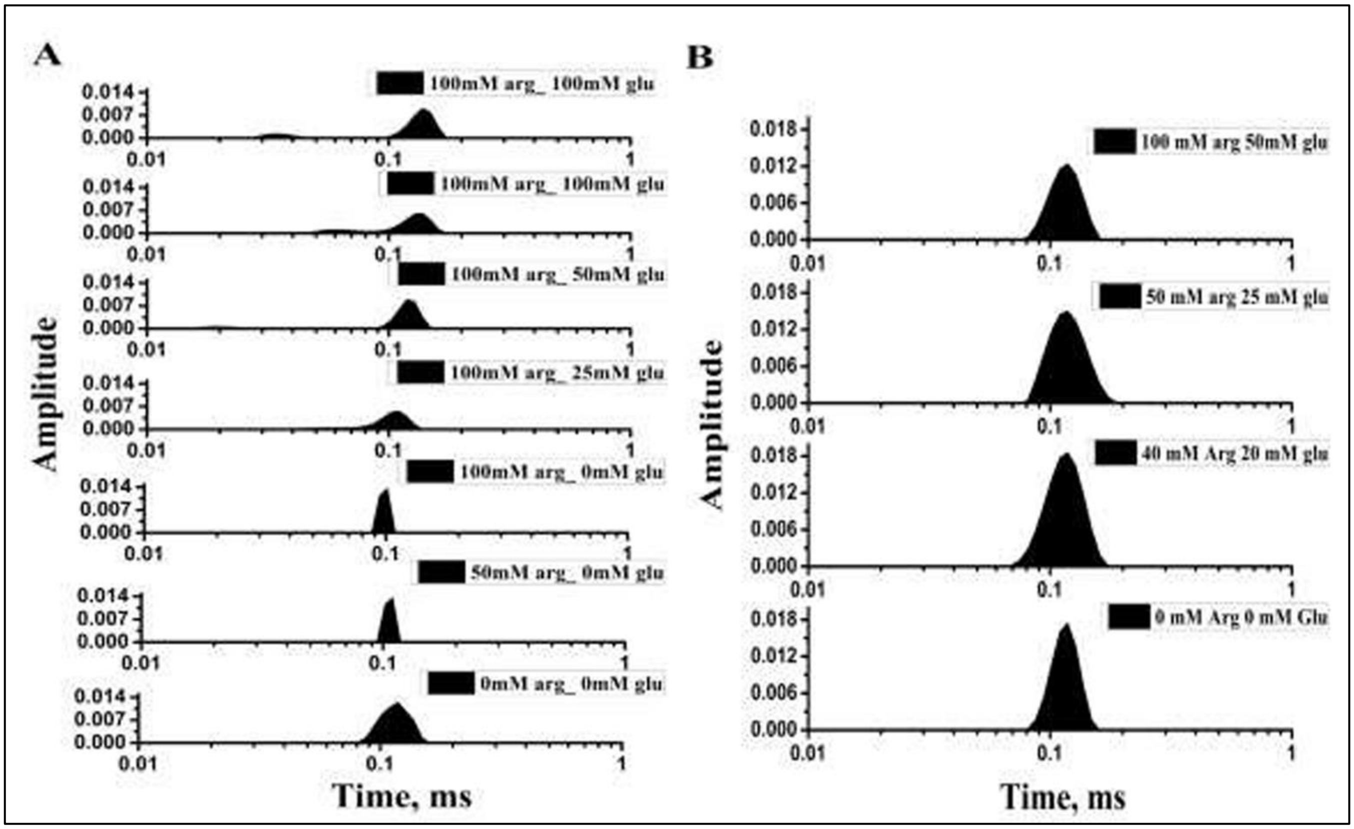

Figure S10: MEM FCS distributions in presence of different concentrations of [A] mixture of Arginine and glutamate and [B] the mixture with a constant 2:1 ratio.

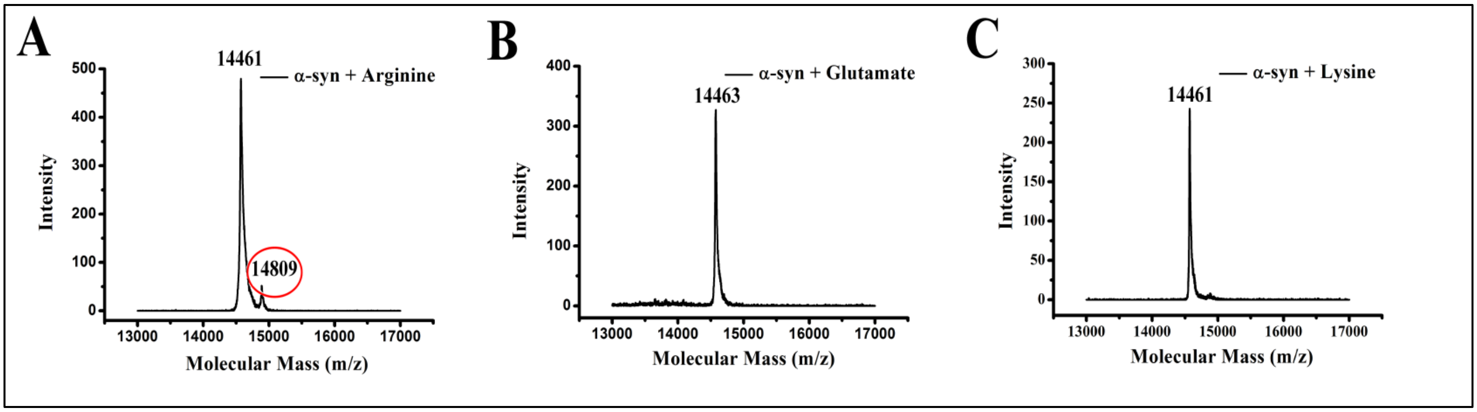

Figure S11: Maldi-TOF profile α-syn with [A] arginine, [B] glutamate, and [C] lysine.
